# Water as a Medicine

**Published:** 1899-06-24

**Authors:** 


### WATER AS A MEDICINE.

In the Baillie Lectures recently delivered at St. George's Hospital, Dr. Howship Dickinson insisted on the important part played by water in man's diet. It constitutes, he said, three-fourths of the solid textures and four-fifths of the blood. It is the indispensable vehicle of all the secretions and of all the materials which are introduced as food. We can, indeed, get on better without meat than without drink. The want of solids is mere deprivation, whereas the want of liquid is this and something more; for, by the lack of water, the escape of what the water should carry off with it is hindered, and the body becomes poisoned by its own products. Thus, when urea and the allied uræmic poisons accumulate in the body as the result of renal disease, water is the surest of eliminants. It cannot leave the body as urine without taking with it more or less of these accumulations. Dr. Dickinson has satisfied himself that the amount of urea excreted in renal disease is increased by the drinking of water in proportion to the amount of diuresis occasioned. One of the symptoms of some forms of chronic and permanent disease of the kidney, particularly of the granular kind, is that the urine is poor in quality, but excessive in amount. The patient is habitually athirst, and drinks abundantly of watery fluids. This may be distressing, but it is his salvation. If he be so ill advised as to greatly reduce his drink the urine too will diminish, and uræmia may declare itself. Again, in diabetes water washes out the sugar and the special toxic material—acetone, or whatever it may be—which the diabetic process engenders, and thus helps to keep the system clear of this poison. In treating this disease if water is stinted the danger is increased, and on the other hand not only may large drinking of water stave off diabetic coma, but this deadly condition may be suspended for a time by the copious introduction of saline solution into the veins. Gout is another disease in which it is probable that the elimination of the essential poison, be it what it may, is facilitated by water drinking and diuresis; and, as Dr. Dickinson adds, "water can be obtained in perfection without going to Germany for it." As to the various minerals which the accidents of geology, rather than any therapeutic purpose, have caused to be mingled with the water at various spas, "these admixtures may do good or may do harm, according to the state of the patient and the judgment of the medical adviser, but the *water* is generally of service, and it may often be a point of wisdom to drink this in its purity rather than sophisticated whether by nature or art."

